# Multi-omics profiling highlights lipid metabolism alterations in pigs fed low-dose antibiotics

**DOI:** 10.1186/s12863-020-00918-3

**Published:** 2020-09-21

**Authors:** Yue Hu, Yihe Zhang, Cong Liu, Rui Qin, Desheng Gong, Ru Wang, Du Zhang, Lianqiang Che, Daiwen Chen, Guizhong Xin, Fei Gao, Qi Hu

**Affiliations:** 1 Genome Analysis Laboratory of the Ministry of Agriculture, Agricultural Genomics Institute at Shenzhen, Chinese Academy of Agricultural Sciences, Shenzhen, 518120 China; 2 Institute of Animal Nutrition, Sichuan Agricultural University, Ya’an, 625014 Sichuan Province China; 3 State Key Laboratory of Natural Medicines, Department of Chinese Medicines Analysis, China Pharmaceutical University, Nanjing, China; 4 Comparative Pediatrics and Nutrition, Department of Veterinary and Animal Sciences, Faculty of Health and Medical Sciences, University of Copenhagen, Frederiksberg, DK Denmark

**Keywords:** Weaned piglets, Low-dose antibiotics, Serum lipidome, Liver methylome, Liver transcriptome

## Abstract

**Background:**

In order to study the relations of hepatocellular functions, weight gain and metabolic imbalance caused by low-dose antibiotics (LDA) via epigenetic regulation of gene transcription, 32 weaned piglets were employed as animal models and randomly allocated into two groups with diets supplemented with 0 or LDA (chlorotetracycline and virginiamycin).

**Results:**

During the 4 weeks of the experiment, LDA showed a clear growth-promoting effect, which was exemplified by the significantly elevated body weight and average daily gain. Promoter methylome profiling using liquid hybridization capture-based bisulfite sequencing (LHC-BS) indicated that most of the 745 differential methylation regions (DMRs) were hypermethylated in the LDA group. Several DMRs were significantly enriched in genes related with fatty acids metabolic pathways, such as FABP1 and PCK1. In addition, 71 differentially expressed genes (DEGs) were obtained by strand-specific transcriptome analysis of liver tissues, including ALOX15, CXCL10 and NNMT, which are three key DEGs that function in lipid metabolism and immunity and which had highly elevated expression in the LDA group. In accordance with these molecular changes, the lipidome analyses of serum by LC-MS identified 38 significantly differential lipids, most of which were downregulated in the LDA group.

**Conclusions:**

Our results indicate that LDA could induce epigenetic and transcriptional changes of key genes and lead to enhanced efficiency of lipid metabolism in the liver.

## Background

Accumulating evidence has shown a strong crosstalk between gut microbiota and host metabolism [1–4], which can provide clues as to how subtherapeutic antibiotics can promote animal growth [5] and they are associated with risks of obesity in prepubertal children [6–8]. In particular, studies have shown that antibiotic exposure can further affect liver functions due to the gut-liver axis [9]. However, genome-scale molecular changes of the liver under antibiotic exposure have not been comprehensively studied.

In recent years, crosstalk between the gut microbiota and host epigenome has also been proposed. Many studies have demonstrated that the products of bacterial fermentation, short chain fatty acids (SCFAs), can affect histone modifications by inhibiting mammalian histone deacetylases (HDAC) [10, 11]. As a major epigenetic mechanism, DNA methylation has been shown to regulate the transcriptional activity of genes and related physiology [12–14]. DNA methylation can also be affected by gut microbiota, considering microbiota-produced substances from gut microbiota, such as folate, cobalamin, pyridoxine, could contribute to the one-carbon metabolism that provides methyl groups. DNA methylation is a key epigenetic mechanism of transcription regulation, which can be affected by microbiota and metabolite changes in the gut, as previously suggested in our and other studies [15, 16]. Considering the gut-liver communications, we hypothesize that the DNA methylome of the liver will be affected upon low-dose antibiotic (LDA) treatment, thereby affecting liver gene expression.

Considering the anatomical and physiological similarities between pigs and humans [17], pigs represent an ideal animal model for studying the growth- and obesity-enhancing effects of antibiotic intervention in infants. In the present study, we aimed to profile the transcriptome and genome-wide DNA methylation of liver tissues in LDA- treated pigs. Along with the analyses of phenotypic data and serum lipidome, we characterized extensive changes in the plasma lipidome, gene expression and promoter methylation in the liver that were elicited by LDA treatment.

## Results

### Growth promotion of LDA- treated piglets

**Fig. 1:**
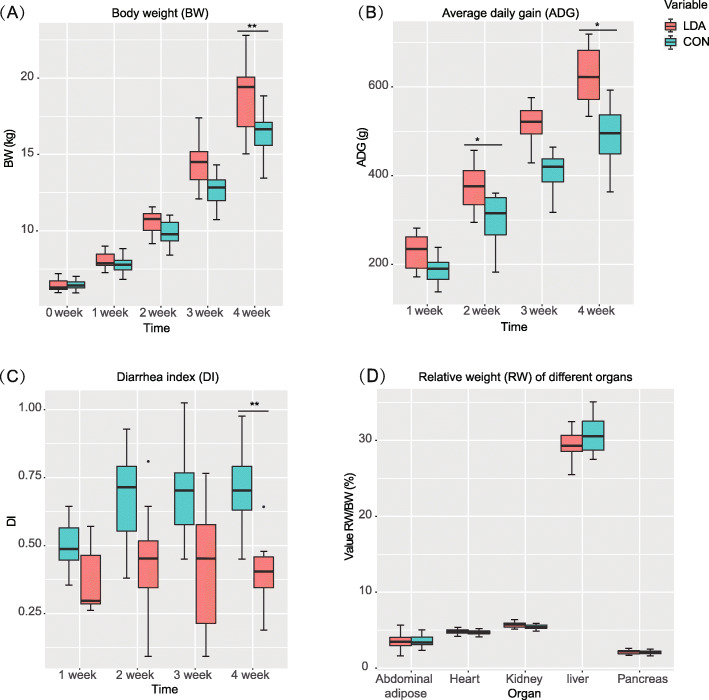
Growth phenotypes of piglets from the LDA and CON groups. Box plots of body weight (**a**), average daily feed intake (**b**), diarrhoea index (**c**) and relative weight (RW) of different organs (**d**) during the 4 weeks of the experimental period between the LDA and CON groups (two-tailed independent t-test, *P-value< 0.05, **P-value< 0.01). RW was obtained from absolute organ weight divided by body weight. The diarrhoea index was expressed as the average daily grade of diarrhoea (three check points per day)

In the present study, we randomly classified 32 Duroc × Landrace × Yorkshire (DLY) female weaned piglets into two groups, either raised with spontaneous microbial colonization as the control (CON) group or treated with two types of broad-spectrum LDA, i.e., chlortetracycline and virginiamycin (see Methods). During the 4 weeks of the experimental period, growth phenotypes and diarrhoea index (DI) [18, 19] for all the DLY piglets were gathered in each week (Table S1) and analysed by SPSS 20.0 (SPSS, Inc.) with a two-tailed independent t-test. During 4 weeks of treatment, a growth-promoting effect was observed starting from the first week, as exemplified by the elevated body weight (BW) and average daily gain (ADG) of the LDA group. In the fourth week, the LDA group showed the most significant difference comparing to the CON group (Fig. 1a, b). In contrast, the occurrence of diarrhoea was significantly decreased in the LDA group (Fig. 1c). On the other hand, there were no obvious differences in the relative weight of different organs between the two groups, suggesting that such growth-promotion was not restricted to specific organs but the whole body (Fig. 1d).

### Serum lipidome analyses

**Fig. 2:**
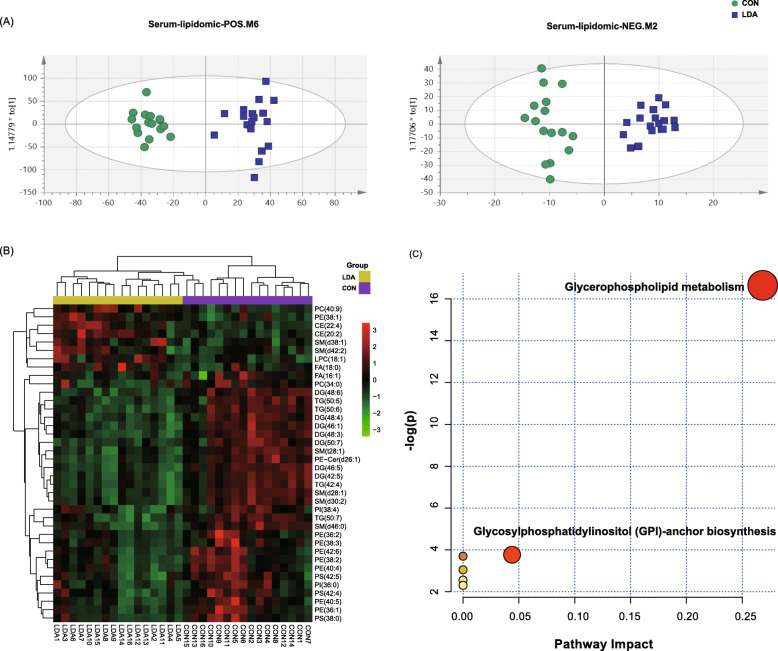
Lipidome comparison showed that altered lipids were mainly involved in glycerophospholipid metabolism. **a** Orthogonal partial least square discriminant analysis (OPLS-DA) of serum lipidome between the CON group (green circle) and LDA group (blue box). **b** Heatmap showing the concentrations of all 38 differential lipids between the two groups. The bars on top of the heatmap display the cluster result of these lipids. **c** KEGG pathway analysis of the differential lipids by MetaboAnalyst (http://www.MetaboAnalyst.ca/)

In addition to growth phenotypes, metabolic parameters from blood were also examined. Total cholesterol (TC) was significantly lower in the LDA group than that in the CON group (Fig. S1), which was analyzed by SPSS 20.0 (SPSS, Inc.) with a two-tailed independent t-test, while the remaining indexes, including alkaline phosphatase (ALP), UREA, glucose (GLU), triglyceride (TG), alanine aminotransferase (ALT), aspartate amino transferase (AST), total bilirubin in serum (TBIL), total bile acid (TBA) and glutamyl transpeptidase (GGT), showed no significant differences (Table S2). Based on this result, we also carried out lipidome analyses of serum by LC-MS in ESI+ and ESI- modes separately. Orthogonal partial least square discriminant analysis (OPLS-DA) was performed to determine the metabolomic distinction between the LDA and CON groups, which showed clear separations of lipid profiles between the two groups (Fig. 2a). Then, a total of 38 significantly differential lipids were identified based on a criterion that the values of variable important in the projections (VIP) were more than 1.0, while the false discovery rate (FDR) -adjusted *P* values were less than 0.05 [20]. Furthermore, these lipids were annotated by the mass of molecular and fragment ions using the database of the lipid maps (http://www.lipidmaps.org/). Consistent with the biochemical analysis of TC, the majority of the differential lipids were significantly downregulated in the LDA group (Fig. 2b, Table S3). Further, KEGG pathway analysis using MetaboAnalyst (https://www.metaboanalyst.ca/) indicated that most of these differential lipids were enriched in the pathways of glycerophospholipid metabolism and glycosylphosphatidylinositol (GPI)-anchor biosynthesis, while fewer were enriched in the pathways of “linoleic acid metabolism”, “alpha linolenic acid metabolism”, “glycine, serine and threonine metabolism”, and “arachidonic acid metabolism” (Fig. 2c, Table S4).

### Liver transcriptome changes in LDA-treated piglets

We then applied a strand-specific mRNA-seq strategy to examine the gene expression profiles of liver tissues from all 32 piglets. A total of 55.4 million paired-end sequencing reads of 150 bp were generated, resulting in 4.1–5.3 gigabyte (GB) of clean sequencing data for each sample with low-quality reads filtered out (Table S5). The clean reads were aligned to the reference genome (*Sscrofa*11.1), with total mapped rates ranging from 85.1 to 89.1% among the 32 samples.

**Fig. 3:**
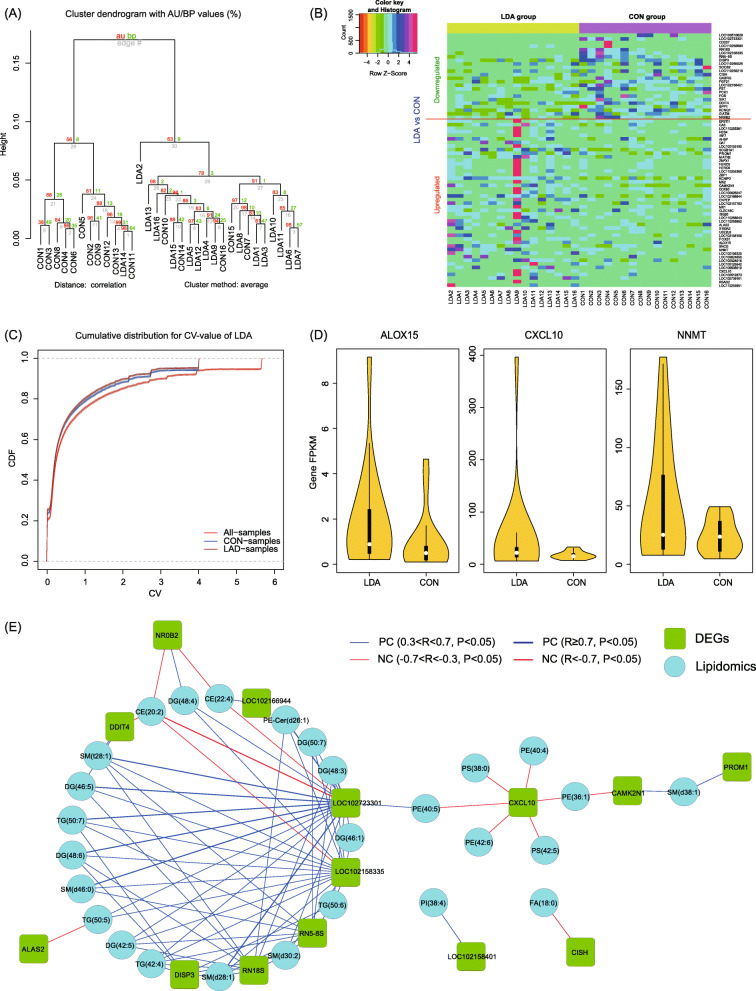
Comparison of the transcriptome of liver samples between the LDA and CON groups. **a** Hierarchical clustering analyses of total gene expression levels from LDA and CON groups by the “Pvclust” algorithm. The approximately unbiased (AU) P-value (%) and bootstrap probability (BP) P-value are shown. **b** Scatter plot of the average expression levels of all DEGs. **c** Covariate analysis between the transcriptome data from the two groups. **d** Violin plots of expression levels from the three key DEGs (ALOX15, NNMT and CXCL10) in the two groups. Black bars indicate the boxplot, and white dots indicate the mean value. **e** Correlation networks of DEGs and differential lipids. Correlation analysis among them is shown in this network. The cyan blocks present DEGs and the green circles are lipids. The blue lines indicate positive correlations and the red lines are negative correlations. And lines with different degrees of thickness indicate different degrees of correlations between these DEGs and lipids

Based on these data, a hierarchical clustering analysis using AU/BP values was performed (distance: correlation, cluster method: average) and most samples were clustered into two root-separated parts according to different groups (Fig. 3a). To clarify which genes were altered, we then performed pair-wise comparisons between the two groups. As a result, differentially expressed genes (DEGs) were obtained under the criterion of |fold change (FC)| > 1.5 and FDR < 0.05 (Fig. 3b, Table S6). Considering that there was clearly a global transcriptomic difference between the two groups, the number of DEGs was only 71 (24 downregulated DEGs and 47 upregulated DEGs compared to the CON group). We then calculated the coefficient of variation for each group, defined as the value of the standard deviation divided by the average. As a result, a high intra-group variation was observed (Fig. 3c), which could explain the limited DEGs. Then these DEGs were put forward to KEGG enrichment analysis using Allenricher [21], which indicated these DEGs were significantly involved in the immune system and metabolic pathways (FDR < 0.05) (Table S7). Moreover, the pathway of glycine, serine and threonine metabolism was also enriched by these differential lipids mentioned above, suggested some kind of regulatory relations between DEGs and differential lipids.

As exemplified by these key upregulated genes, the liver transcriptome analyses indicated highly elevated functions of lipid biosynthesis and metabolism for these LDA-treated piglets. Despite this complication, among these DEGs, we found two significantly differential genes, ALOX15 (log2(FC) = 1.05635, FDR = 0.007295) and NNMT (log2(FC) = 1.09953, FDR = 0.007295), that were highly elevated for expression in the LDA group. Both genes function in lipid metabolism, especially the ALOX15 gene, whose encoded enzyme (lipoxygenase) acts on various polyunsaturated fatty acid substrates to generate various bioactive lipid mediators [22]. CXCL10 (log2(FC) = 1.4118, FDR = 0.007295), an important antimicrobial gene, was also significantly upregulated in the LDA group (Fig. 3d). The alterations of gene expression in these important DEGs indicated an important role in the regulation of lipid metabolism elicited by LDA treatment. Besides, we used quantitative RT-PCR (qRT-PCR) to validate the mRNA expression levels of these three key genes, and the results indicated significant upregulation in the LDA group compared to the CON group (Fig. S2).

We further examined the correlation between DEGs fpkm and differential lipids abundance from all the samples. As a result, we found that there were several DEGs have complex correlations with these differential lipids (Fig. 3e). Especially for the CXCL10, which was an antimicrobial gene and significantly up-regulated expression in LDA group mentioned above, had negative correlations with two kinds of glycerophosphoethanolamines (PE) and four kinds of glycerophosphoglycerols (PS). Furthermore, the followed correlated gene, CAMK2N1, was also involved in immunity and acted as a tumor suppressive role in prostate cancer cells [23]. It was suggested that these lipids may play negative roles in immunity. As to other DEGs, such as DISP3, has positive correlations with several kinds of triacylglycerols (TG) and diradylglycerols (DG). Moreover, DISP3 encodes a sterol-sensing domain-containing protein that links cholesterol metabolism [24], which further implied the close correlation of DISP3 and lipid metabolism.

### Genome-wide liver DNA methylome

**Fig. 4:**
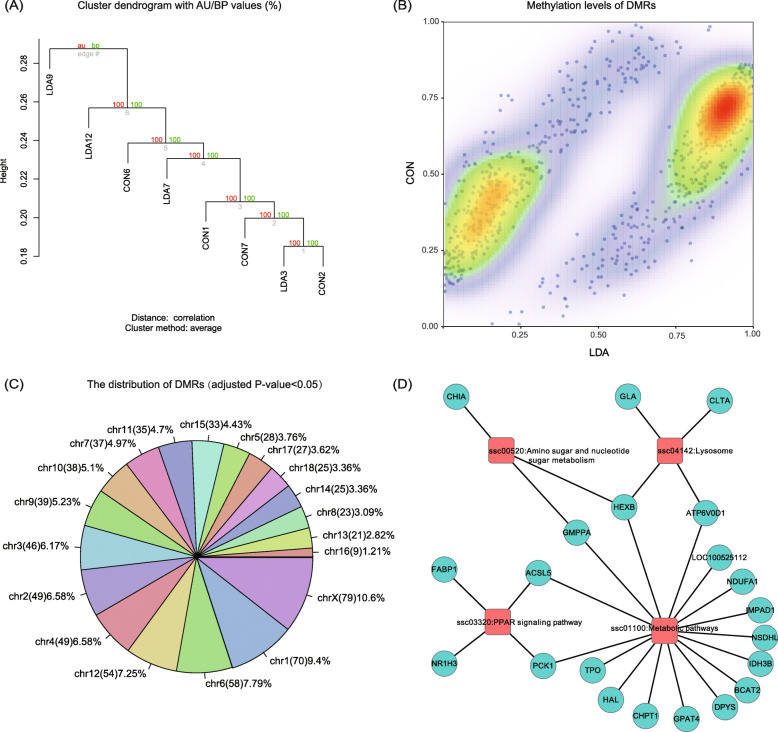
Comparison of promoter methylome of liver samples between the LDA and CON groups. **a** Hierarchical clustering analysis of the average DNA methylation levels in promoter regions by the “Pvclust” algorithm. The approximately unbiased (AU) P-value (%) and bootstrap probability (BP) P-value are shown. **b** Scatter plot of the average methylation levels of DMRs in the LDA and CON groups. **c** The pie chart showing the distribution of DMRs in different chromosomes. **d** KEGG pathways enriched by the DMR-associated genes

To thoroughly profile the genome-wide DNA methylation of pigs in a more cost-effective way, we adapted a liquid hybridization capture-based bisulfite sequencing (LHC-BS) approach [25, 26], whose efficacy has previously been comprehensively demonstrated in the human genome. Based on NCBI Refseq gene annotation of the pig reference genome (*Sscrofa*11.1), the gene promoters were denoted as regions from upstream 2000 bp to downstream 1000 bp of the transcriptional start sites (TSS). A total of 32,163 capture probe regions with a total length of 156.1 MB were customized, which enabled the coverage of 21,234 genes (69.98% of the total RefSeq genes) in the NCBI database. We then profiled the promoter methylome of 8 liver samples (see Methods). We generated an average of 14.6 GB clean data for each sample, reaching a mean sequencing depth of 37 ×, with an average bisulfite conversation rate of 98.77%. The BSMAP algorithm [27] was then applied to align the sequencing reads back to the reference genome, resulting in 58.5% of the sequencing reads being uniquely mapped. As a result, on average, 6.47 million CpG sites and 72.4 million non-CpG sites were covered for at least 5× depth (Table S8). Then, a hierarchical clustering analysis was performed to examine the overall methylation status of the whole genome based on commonly covered cytosine sites across the eight liver samples (Fig. 4a). The two groups were not clearly separated, probably owing to small sample size and individual epigenomic variations. Nevertheless, gene-specific methylation divergence may be revealed between the two groups. Therefore, we carried out comparisons between the two groups to screen for DMRs. A scatter plot on the average levels of these DMRs showed that most DMRs were hypermethylated in the LDA group (Fig. 4b). This approach generated a total number of 745 DMRs (FDR < 0.01), that were extensively distributed across the whole genome (Fig. 4c). Based on Refseq gene annotation of the pig genome, 714 DMR-associated genes (DMRGs) were identified, including genes containing DMRs both in their promoters or gene bodies (Table S9). KEGG analyses indicated that key genes involved in metabolic pathways were significantly enriched, including FABP1 and PCK1 in the PPAR signaling pathway (Fig. 4d).

## Discussion

Antibiotic usage represents a major concern in global public health. Numerous pieces of evidence have indicated that overuse of antibiotics is linked to adverse health conditions such as obesity, inflammation and host-microbe imbalance [28–30]. In particular, even brief antibiotic treatments can have long-term effects on microbiota composition, while changes in the gut microbiome may affect the energy harvest from diet and energy storage and expenditure through fatty acid oxidation [31]. Dysbiosis has been associated with many disease states including autoimmune diseases, metabolic diseases, and malnutrition. Therefore, alterations to microbiota compositions caused by antibiotics are likely to have additional health consequences, specifically associated with weight gain and metabolic imbalance, as well as susceptibility to diseases. Indeed, early childhood antibiotic exposure is associated with an increased risk for excessive weight gain, allergies and inflammatory bowel diseases (IBD) [32, 33].

Based on previous observations, we hypothesize that the liver epigenome could be affected by the intestinal microbiome through the gut-liver axis, which may further affect liver cellular functions by epigenetic transcription regulation. Previously, many gut microbiota studies were based on rodent models [34, 35], because their complete microbiota modulation is more easily manipulated, either by germ-free rearing following inoculation with specific gut microbiome or treatments with long-term and broad-spectrum antibiotics. In our study, pig was used as the animal model for its high similarities with humans in many aspects, such as metabolism and body development [36, 37], as well as gut microbiota [15]. We treated these piglets with LDAs (chlortetracycline and virginiamycin), resulting in significantly promoted host growth. We then intended to comprehensively profile the DNA methylome and transcriptome of liver tissues as well as the serum lipidome, to fully understand the effects of LDA on host metabolism that could lead to growth promotion and even overweight or obesity.

After a four-week experimental period, phenotypic differences were present between the LDA and CON groups through data analysis by SPSS 20.0 (SPSS, Inc.) and the TC level was significantly downregulated in the LDA group. Next, to guarantee an unbiased screening, we first put forward to untargeted lipidome detection of serum from the two groups, and then we adapted applied strand-specific RNA-seq technology, which provides a more accurate estimate of transcriptional expression compared to commonly use non-stranded RNA-seq methodology [38]. Regarding the pig liver DNA methylome, we used the LHC-BS approach [25] to profile the single-base-pair resolution promoter methylome. Although diverse technologies have been developed in the past decades, target- capture represents a more cost-effective and comprehensive approach of genome-wide analysis.

As a result, our results have shown considerable alterations of the transcriptome and DNA methylome of liver, as well as the serum lipidome, which mainly highlighted molecular changes of genes functioning in lipid metabolism of these piglets. The lipidome analysis results suggested the whole tendency of downregulated differential lipids in the LDA group compared to the CON group. For the liver transcriptome analysis, there are three key DEGs (ALOX15, CXCL10 and NNMT) with the function of lipid metabolism and immunity. ALOX15, as one of the various LOX-isoforms, exhibits multiple catalytic activities. They oxygenate polyenoic fatty acids to hydroperoxy derivatives but also exhibit lipohydroperoxidase activity (sometimes also called hydroperoxide isomerase activity), which converts lipid hydroperoxides to secondary lipid peroxidation products [39]. 15-LOX-1 also plays a major role in the formation of arachidonic anti-inflammatory products, known as lipoxins [40], which suggested its regulatory role in the immune system. Studies show that the ALOX12/15 family of enzymes and their pro-and anti-inflammatory metabolites in obese humans with and without type 2 diabetes (T2D). ALOX12 expression is positively correlated with the expression of CXCL10 [41], which is a key antimicrobial gene that is involved in the TNF signaling pathway. In addition, increasing NNMT expression in the liver could stabilize the Sirtuin 1 protein, an effect that is required for glucose and cholesterol metabolism to decrease the levels of serum and liver cholesterol and liver triglycerides in mammals [42, 43]. Therefore, upregulated gene expression level of NNMT in the liver could be the reason for the lower concentrations of serum lipids in the LDA group, which might be more beneficial for maintaining homeostasis of glycerophospholipids in mammalian cells and even better for body growth and development [44]. However, limitation of our present study might be that we used a limited number of samples, especially for methylome analysis. The complexity of the pig genome might also be the reason for high intra-group individual variations, which blurred the real differences.

Additionally, methylome analyses indicated that dozens of genes containing DMRs were significantly enriched in metabolic pathways, including the FABP1 gene in the PPAR signaling pathway, which encodes fatty acid binding protein that binds LCFAs and other hydrophobic ligands. Studies have indicated that FABP1 is essential for proper lipid metabolism in differentiated enterocytes, particularly concerning fatty acid uptake and its basolateral secretion [45]. Additionally, the PCK1 gene, which is involved in both the PPAR signaling pathway and metabolic pathway, is a main control point for the regulation of gluconeogenesis. The expression of this gene can be regulated by insulin, glucocorticoids, glucagon, cAMP, and diet and it has multiple relationships with several metabolic diseases [46, 47].

## Conclusions

In conclusion, our results still provide preliminary evidence that LDA treatment could induce epigenetic changes in key gene pathways, especially the lipid metabolism pathway, which further induced transcriptional alterations in liver tissue. These important functional DEGs (ALOX15, CXCL10 and NNMT) related to lipid metabolism and immunity are highly correlated with the serum lipidome results, which showed significantly decreased levels of lipids in the serum. Together with the highly promoted growth phenotype, we can conclude that LDA- treatment has induced a systematic genome-level of changes in liver tissues and contributed to a more efficient metabolism of lipids.

## Methods

### Pig model and sample collections

Thirty-two female DLY piglets (without litters) were supplied by Sichuan Agricultural University and raised in their hog house with the temperature control at 23–25 °C, where the environments and production performance were stable and the feeding management was standardized. These piglets were randomly allocated into two groups with diets supplemented with 0 or low-dose of antibiotics (CON vs. LDA), which were kept separately in two different pens of the hog house. Two piglets in the same group were kept in one cage and were fed 4 times a day (8:00, 12:00, 16:00 and 20:00), and the feeding status of piglets was checked every 2 h. The feeding trough was a rectangular four-mouth long trough, and the drinking water tank was the nipple type. All the piglets were fed and drank freely and weighed with an empty stomach every Monday morning during the entire experimental period of 4 weeks.

Two types of antibiotics were mixed in the fodder according to government standards (chlortetracycline was mixed in fodder with a dose of 75 g/1000 kg and virginiamycin was 25 g/1000 kg). These two groups of piglets were fed separately and the LDA group were fed with LDAs at age of 21 ± 2 days. The feedings were continuous for 4 weeks, with the CON group fed with the normal fodder at the same time. Blood samples were collected 1 day before euthanasia and serum was separated by centrifugation at 3500 rpm for 15 mins and then kept at 4 °C. At the end of the experimental period, all of these piglets were intravenously injected with pentobarbital sodium (15 mg/kg of BW) before jugular exsanguination. After the abdomen was exposed, the abdominal adipose, lung, liver, spleen, heart, kidney, stomach, small intestine colon, and pancreas were quickly resected and their wet weights were recorded. Small liver sample pieces were collected and saved at − 80 °C after being snap-frozen in nitrogen for the subsequent experiments, as well as tissues from other organs for more research.

### Metabolic parameters

The detections of metabolic parameters, such as the serum concentrations of ALP, urea, GLU, TG, ALT, AST, TC, TBIL, TBA and GGT were performed based on the previous study [48], with an automatic biochemical analyser (Model 7020, Hitachi, Tokyo, Japan) and corresponding commercial kits (Sichuan Maker Biotechnology Inc., Chengdu, China). There was less than 5% variation between the intra-assay and inter-assay coefficients for each assay.

### Serum lipidome analysis

Internal standards were dissolved by DCM: MeOH (2:1, v/v, 0.1% BHT) and mixed at the final concentration of 30 μg/ml FFA (17:0), 40 μg/ml LPC (17:0), 70 μg/ml PC (17:0/17:0) and 300 μg/ml TG (17:0/17:0/17:0). For the preparation of lipid extracts, 30 μl thawed serum samples were mixed with 30 μl of the internal standard mixture, followed by 540 μl DCM: MeOH (2:1, v/v, 0.1% BHT) and then vortexed thoroughly. After adding 100 μl of H_2_O, 100 μl of the lower organic phase was collected by centrifuging at 14,000 g for 10 min, and samples were reconstituted with 1 ml ACN: IPA (1:1, v/v) before UHPLC-Orbitrap analysis. For method validation, quality control (QC) samples were prepared by pooling small aliquots of serum samples to ensure broad metabolite coverage. The precision was determined by intra- and inter-day variability. For the UHPLC conditions, the injection volume was 2 μl for each sample and the column was a waters ACQUITY BEH C18 column (2.1 × 100 mm, 1.7 μm) with a temperature of 55 °C. The index of the mobile phase was set as follows: solvent A: 40:60 water/ACN (0.1% formic acid and 10 mM ammonium acetate, only in positive ion mode), solvent B: 90:10 IPA/CAN. The gradient was 0–13 min 30–80% B; 13–20 min 80–90% B; 20–21 min 90–100% B; 21–26 min 100% B; re-equilibrate: 10 min, with a flow rate of 200 μl/ml. These raw data were subjected to SIEVE 2.1 software (Thermo Fischer Scientific) for pretreatment. Finally, three- dimensional matrices consisting of sample information, peak intensity, retention time (RT) of peak and the mass-to-charge ratio (m/z) were obtained. Multivariate statistical analysis was performed using SIMCA-P + 14.1 (Umetrics, Umeå, Sweden) software with the results of OPLS-DA. Differential lipids were selected based on the comparisons between the LDA and CON groups with the filter conditions of VIP > 1 and *P*-value< 0.05. KEGG analysis of these differential lipids was performed by MetaboAnalyst (https://www.metaboanalyst.ca/).

### Library construction for strand-specific mRNA sequencing

RNAs were extracted by TRIzol and quantified on a Qubit®3.0 fluorometer (Thermo Fisher Scientific, Cat#Q33216). A total amount of 1.5 μg of RNA per liver sample was used as input material for preparations. Sequencing libraries were constructed using the NEBNext® UltraTM RNA library Prep Kit for Illumina® (NEB, USA) following the manufacturer’s recommendations. In brief, mRNA was purified from the total RNA using poly-T oligo-attached magnetic beads. Fragmentation was carried out using divalent cations under elevated temperature in NEBNext First Strand Synthesis Reaction Buffer (5×). First strand cDNA was synthesized using a random hexamer primer and M-MuLV Reverse Transcriptase (RNaseH). Second strand cDNA synthesis was subsequently performed using DNA polymerase I and RNaseH, with the dUTP instead of dTTP. The remaining overhangs were converted into blunt ends via exonuclease/polymerase activities. After adenylation of the 3′ ends of DNA fragments, NEBNext Adaptor with hairpin loop structure was ligated to prepare for hybridization. To select cDNA fragments of the right length, the library fragments were purified with an AMPure XP system (Beckman Coulter, Beverly, USA). Then 3 μl of USER Enzyme (NEB, USA) was incubated with size-selected, adaptor-ligated cDNA at 37 °C for 15 min followed by 5 min at 95 °C before PCR, which allowed selective degradation of the second cDNA strand containing dUTP for the reservation of the transcript direction and strand-specific information. PCR was then performed with Phusion High-Fidelity DNA polymerase, universal PCR primers and index primers. Finally, the products were purified (AMPure XP system), and the library quality was assessed on the Agilent Bioanalyzer 2100 system. The clustering of the index-coded samples was performed on a cBot Cluster Generation System using a HiSeq4000 PE Cluster Kit (Illumina) according to the manufacturer’s instructions. After cluster generation, the library preparations were sequenced on an Illumina Hiseq4000 platform and 150 bp paired-end reads were generated.

### Strand-specific mRNA data analysis

The raw reads of the 32 RNA samples were processed by removing the adaptor sequences and low-quality sequences (low quality threshold (default [5]), low quality rate (default [0.5]), N rate threshold (default [0.05]), and PCR duplications were removed. Clean reads were aligned to the pig reference genome (*Sscrofa*11.1) using Tophat2 (Version 2.0.12) [49]. Next, the Cufflinks (Version 2.2.1) tool was used to quantify transcript abundance in terms of fragment per kilobase (Kb) of exon model per million mapped fragment (FPKM) following the default options, and added the -G/−−GTF -guide, which was quantitated against reference transcript annotations (*Sscrofa*11.1). FPKM of each sample was counted to estimate the expression levels of the transcripts by the Cuffdiff package [50]. The analysis was conducted using a binomial test on variance estimated and size factor normalized data [51]. All obtained *P*-values were adjusted for FDR due to multiple testing procedures used to control for type I error [52, 53]. Finally, we used |FC| > 1.5 and FDR < 0.05 to identify genes with significantly expressed changes in liver samples from the CON group versus the LDA group.

### LHC-BS library construction and sequencing

To ensure the power of statistical analysis, 4 samples were randomly selected in each group (8 samples in total) for LHC-BS library construction. DNA from the liver tissues from these piglet samples was extracted by the DNeasy Blood & Tissue Kits (QIAGEN, Cat#69504) according to the manufacturing instruments and quantified by a Qubit®3.0 fluorometer (Thermo Fisher Scientific, Cat#Q33216). Promoter-targeted LHC-BS was performed as previously described [25]. Briefly, 1 μg of DNA per sample was processed by fragmentation using Covaris E210 Ultrasonicator (Covaris, Inc., 294,448), followed by blunt end repair, 3′-adenylation, and 5′-methylcytosine index adapter ligation. Then, 250 ng of DNA from each of three/four adaptor-ligated libraries was pooled together for the liquid hybridization capture procedure. The hybridization probe was synthesized and purchased from Roche Nimblegen Incorporation. Finally, the captured DNA was eluted in 50 μl of 10 M NaOH with incubation at room temperature for 10 min. The supernatant was transferred into a new tube and neutralized with 50 μl of 10 M HAc and then purified using a MiniElute PCR Purification Kit (QIAGEN, Cat#28004). For bisulfite conversion, 200 ng of unmethylated λDNA was added into each captured product and then ZYMO EZ DNA Methylation-Gold Kit™ (ZYMO, Cat#D5005) was employed to convert unmethylated cytosine into uracil according to the instructions. After purification, PCR was carried out with JumpStart™ Taq DNA Polymerase (Sigma, Cat#D9307) using the program of 94 °C for 30s, 15 cycles of 94 °C for 10 s, 60 °C for 30 s, 72 °C for 40s then prolonged at 72 °C for 5 min and held at 12 °C. The PCR products were purified using AMPure XP beads (Agencourt, Cat#A63881) and were quantified by the Agilent Bioanalyzer 2100 system (Agilent Technologies, CA, USA). After the qPCR assays, the LHC-BS libraries were sequenced by the Illumina Hiseq Xten platform with a sequencing strategy of paired-end 150 base pairs.

### Data analysis of promoter-targeted LHC-BS

After removing the adapter sequences and filtering out the low quality reads, the LHC-BS sequencing data were directly aligned to the pig reference genome (*Sus scrofa*11.1) using BSMAP 2.73 [27]. The DNA methylation level of a specific cytosine was then calculated as the number of reads supporting methylation divided by the total number of reads covering that cytosine. DMRs were identified by metilene [54] with usage of the sliding window strategy: commonly covered CpG sites with sequencing depth ≥ 5X between two groups of samples were selected as candidate sites. Then, the first CpG with significantly differential methylation (*P*-value < 0.05) was used as an initial locus of DMR, and the candidate sites were merged into a candidate DMR according to the following criteria: the distance between two neighbouring candidate CpG sites ≤300 bp; all the candidate CpG sites in the candidate DMR maintain the same methylation direction (hyper- or hypo-); a candidate DMR must harbour at least 5 candidate CpG sites; for each of the above candidate DMRs, a chi-square test was performed to filter out the regions with a P-value > 0.05 and average methylation levels between two samples < 20%. Pvclust was used to perform hierarchical cluster analysis via function hclust in R [55], and for each cluster in hierarchical clustering, quantities called *P*-values were calculated via multiscale bootstrap resampling.

### Quantitative RT-PCR (qRT-PCR)

Total RNAs were extracted by TRIzol and quantified on a Qubit®3.0 fluorometer that mentioned above. For cDNA synthesis, 400 ng of RNA was reversed transcribed using the RevertAid First Strand cDNA Synthesis Kit (Thermo Fisher Scientific, Cat#K1621). qRT-PCR was performed using SuperReal Premix Plus (SYBR Green) (TIANGEN, Cat#FP205) on a StepOnePlus™ Real-Time PCR System (Thermo Fisher Scientific, Cat#4376600) using 96-well optical reaction plates. Samples from seven piglets per group were analyzed and all the PCR primers were designed using the Primer Premier 5.0 software (PREMIER Biosoft, CA), which were listed in Table S10. Relative gene expression values were calculated by the comparative CT (threshold cycle) method (ΔΔCT method, Applied Biosystems) [56]. The comparative CT method gives the amount of target gene normalized to an endogenous reference gene (GAPDH) and to a relative calibrator sample. Data analysis and statistics were performed by Wilcoxon test and data with *p*-value < 0.05 were considered statistically significant.

### Correlation analysis

Correlations between DEGs and differential lipids have an absolute Pearson’s correlation above 0.50 with a significance level under 0.05, and these correlations were transformed into links between genera and SCFAs in the co-occurrence network using self-develop perl script. The co-occurrence networks were then visualized using Cytoscape 2.8.3.

## Supplementary information

**Additional file 1: Figure S1.** Comparison of total cholesterol (TC) between the LDA and CON groups.

**Additional file 2: Figure S2.** Validation of mRNA expression levels of three key DEGs by qRT-PCR.

**Additional file 3: Table S1.** Phenotypic (productive) data of all the DLY piglet samples. **Table S2.** Summary of metabolic parameters in serum samples. **Table S3.** Differential lipids between the antibiotic and control group. **Table S4.** Pathway analysis results of these differential lipids by MetaboAnalyst 4.0. **Table S5.** Data summary of liver transcriptome. **Table S6.** DEG information by transcriptome data processing. **Table S7.** KEGG enrichment analysis of DEGs. **Table S8.** Liver methylome data processing. **Table S9.** DMRs on different chromosomes based on LHC-BS data processing. **Table S10.** Primer used in quantitative RT-PCR.

## Data Availability

The RNA-seq and LHC-BS data analysed during the current study are deposited in the GEO (Gene Expression Omnibus) database under the accession number GSE122027.

## Abbreviations

LDA: Low-dose antibiotics
LHC-BS: Liquid hybridization capture-based bisulfite sequencing
CON: Control
DMRs: Differential methylation regions
DMRGs: DMR-associated genes
DLY: Duroc × Landrace × Yorkshire
SCFAs: Short chain fatty acids
HDAC: Histone deacetylases
BW: Body weight
ADG: Average daily gain
RW: Relative weight
TC: Total cholesterol
ALP: Alkaline phosphatase
GLU: Glucose
TG: Triglyceride
ALT: Alanine aminotransferase
AST: Aspartate amino transferase
TBIL: Total bilirubin in serum
TBA: Total bile acid
GGT: Glutamyl transpeptidase
OPLS-DA: Orthogonal partial least square discriminant analysis
VIP: Variable important in the projections
GPI: Glycosylphosphatidylinositol
GB: Gigabyte
DEGs: Differentially expressed genes
TSS: Transcriptional start sites
IBD: Inflammatory bowel diseases
T2D: Type2 diabetes
QC: Quality control
RT: Retention time
m/z: Mass-to-charge ratio
RNaseH: Reverse transcriptase
FPKM: Fragment per kilobase (Kb) of exon model per million mapped fragment
FDR: False discovery rate
FC: Fold change
qRT-PCR: Quantitative RT-PCR
