# Supplementary figures and images for: Multi-omics profiling highlights lipid metabolism alterations in pigs fed low-dose antibiotics
Source: BMC Genet. 2020 Sep 21;21:112. doi: 10.1186/s12863-020-00918-3 (PMC7507292; doi:10.1186/s12863-020-00918-3)

**S1 Fig**

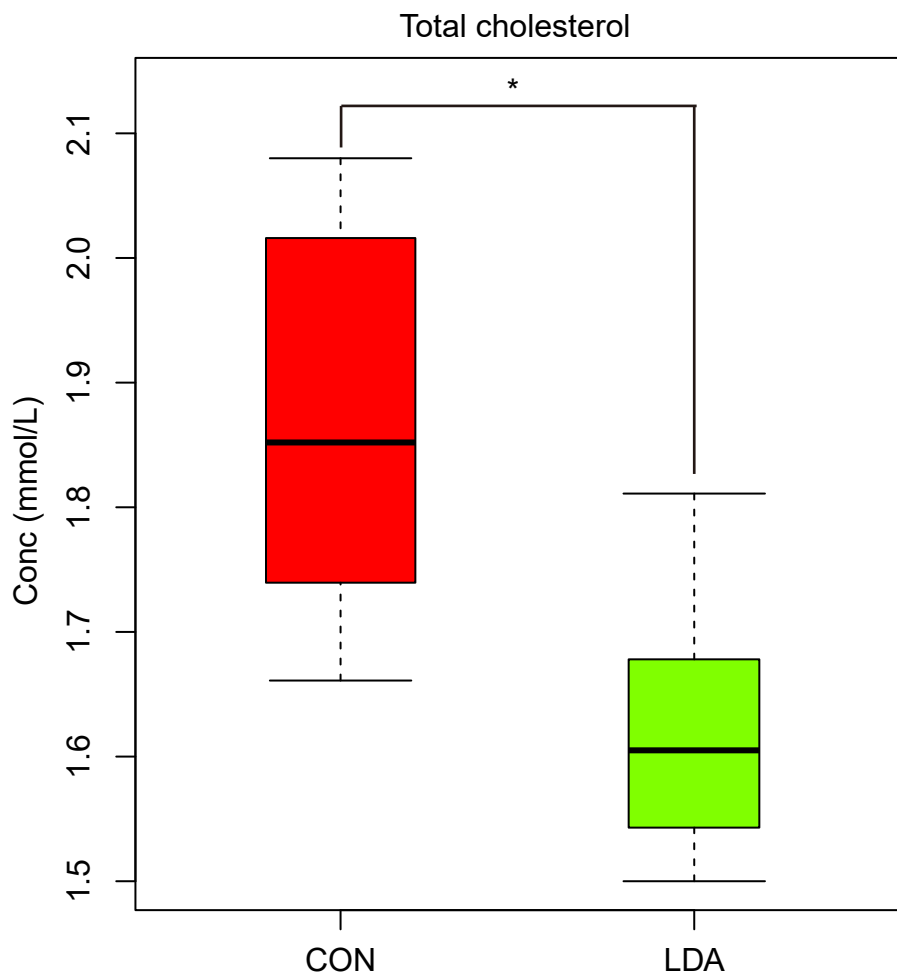

(\*P-value<0.05)

Supplement: Supplementary file 1 — Additional file 1: Figure S1. Comparison of total cholesterol (TC) between the LDA and CON groups. [file 12863_2020_918_MOESM1_ESM.pdf]

**S2 Fig**

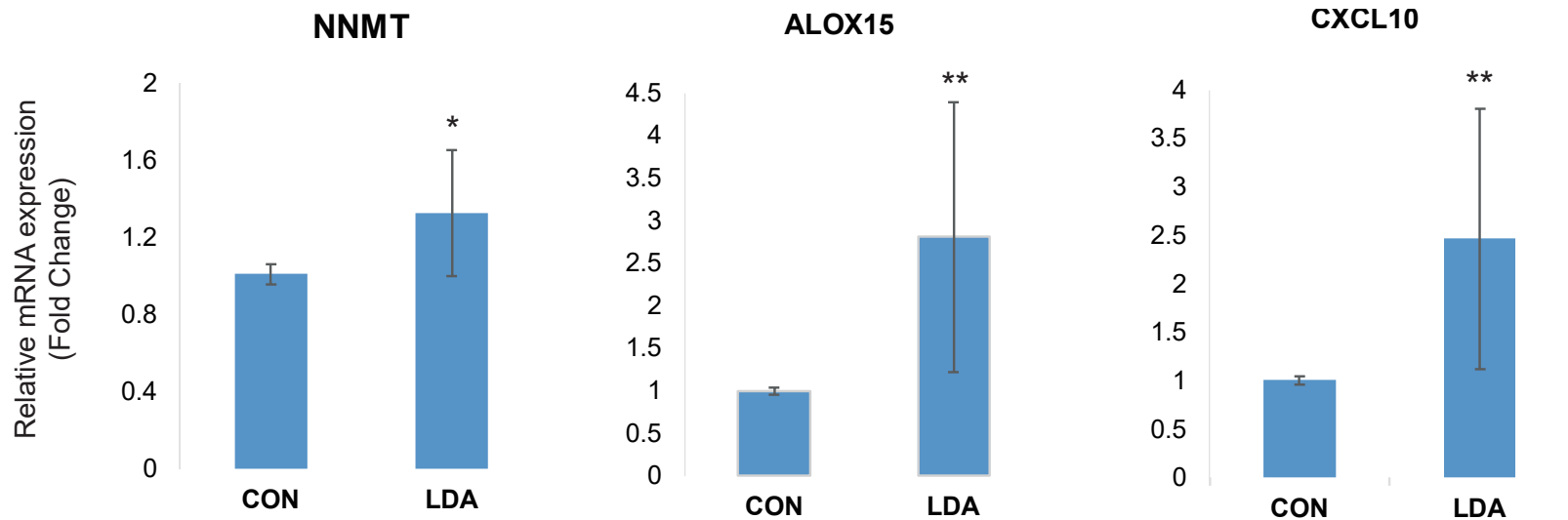

(\*P-value<0.05, \*\*P-value<0.01)

Supplement: Supplementary file 2 — Additional file 2: Figure S2. Validation of mRNA expression levels of three key DEGs by qRT-PCR. [file 12863_2020_918_MOESM2_ESM.pdf]
